# Sensory Modulation and Sleep Quality among Adults with Learning Disabilities: A Quasi-Experimental Case-Control Design Study

**DOI:** 10.1371/journal.pone.0115518

**Published:** 2015-02-06

**Authors:** Kineret Sharfi, Sara Rosenblum

**Affiliations:** The Laboratory of Complex Human Activity and Participation, Department of Occupational Therapy, Haifa University, Haifa, Israel; Cognitive Brain Research Unit, FINLAND

## Abstract

**Purpose:**

Following the International Classification of Functioning, Disability and Health (ICF) concepts, this study examines body functions such as sensory modulation and sleep quality among adults with learning disabilities (LD).

**Methods:**

One hundred and ten participants, 55 adults with LD and 55 matched controls (mean age 30 years) filled in a socio-demographic questionnaire, the *Adults/Adolescents Sensory Profile* (AASP), and the *Mini Sleep Questionnaire* (MSQ). Chi-tests, Mann-Whitney tests, and Kolmogorov-Smirnov tests were conducted to examine group differences related to socio-demographic characteristics and body functions. Correlation and regression analyses were conducted to examine relationships between body functions.

**Results:**

Significant differences were found between the groups in: (a) unique socio-demographic variables: high-schools attended, family status and number of children; (b) body functions: low registration and sensory sensitivity (p < .001), sensory avoiding (p = .002), sensory seeking (p = .021) and sleep quality (p < .001). Significant correlations were found between AASP subscale scores and the MSQ final score in each group. Regression analysis revealed that for the entire sample (N = 108), low registration accounted for 10.2% of the variance of sleep quality above group membership (p < .001), while in a separate examination of adults with LD (n = 53), low registration accounted for 19.9% of the variance of sleep quality (p < .001).

**Conclusions:**

Adults with LD need to be studied through a health-related perspective such as the ICF model to gain further understanding of their unique characteristics and daily needs. Sensory and sleep functions of adults with LD should be further studied in the context of health related quality of life.

## Introduction

### Learning disabilities

Learning disabilities (LD) is a term for a large group of neurological disorders caused by deficits in the central nervous system which influence the individual’s ability to efficiently maintain, process or convey information to others [1]. The up to date common definitions of LD [2, 3] focus on deficient academic skills [4] such as the imperfect ability to listen, think, speak, write, spell, or perform mathematical calculations [5] and therefore LD are usually diagnosed within the educational system [4, 6]. However, literature reveals that in addition to their academic difficulties, children, as well as adolescents with LD have to deal with social and emotional difficulties [7, 8].

LD frequently co-occur with other health conditions appearing in the Diagnostic and Statistical Manual of Mental Disorders (DSM-IV/5) [2, 9]. The data regarding associated health conditions can be gathered from assorted literature on children, adolescents and adults with LD. For example, between 25% - 50% of children faced with LD were reported as dealing with Attention Deficit Hyperactivity Disorder (ADHD) as well [10]; at least 50% of children with LD were identified with concomitant Developmental Coordination Disorder (DCD) [11]; depression and anxiety disorders were reported in 40% - 50% of this population [12, 13].

The complicated clinical picture of the population with LD has led some investigators to suggest using the term Atypical Brain Development (ABD) to include people with specific LD such as reading, writing, spelling and arithmetic deficits as well as DCD, ADHD and Specific Language Impairment (SLI) [14]. Others suggested reuniting the various developmental disorders and emphasizing the diagnostic significance of ‘secondary’ symptoms [15].

LD have been identified continuing into adulthood, with the daily experience not necessarily centered on the academic domain [16]. Literature reveals that adults with LD experience difficulties in employment [17] and in activities of daily living, including organization, banking, and time and home management [18]. Additional difficulties in friendships and dating, partnerships, parenting, and general perception of quality of life (QoL) are reported in qualitative literature [19]. Even so, there is still a paucity of evidence-based research to guide research and practice regarding adults with LD and a conceptual framework for studying their diverse life outcomes is still missing [16].

### The International Classification of Functioning, Disability and Health

Due to the complexity of various health conditions among the population with LD as well as evidence for difficulties in additional life areas, this article suggests the use of a comprehensive health perspective such as the International Classification of Functioning, Disability and Health (ICF) model [20] in order to gain further understanding of the complicated health characteristics and daily needs of adults with LD.

**Fig 1:**
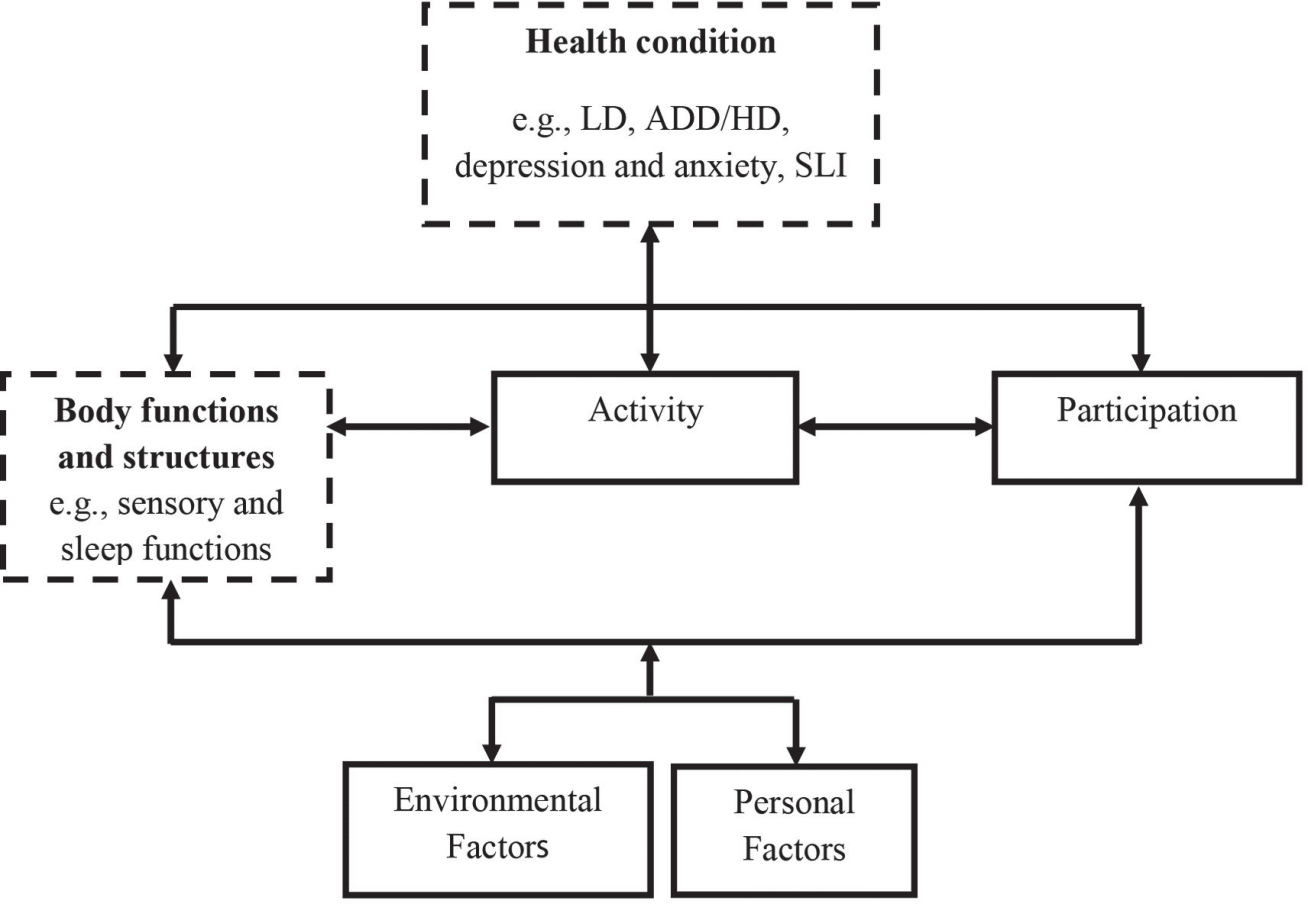
Components of the ICF model (WHO, 2001).

The ICF framework presented by the World Health Organization includes the following components as its central concepts: health condition, contextual factors, body functions and structures, activity and participation [20] (Fig. 1). Levels of activity and participation are viewed as outcomes of interactions between health conditions and contextual factors. Health condition refers to any disease, disorder or injury that exists in a person’s life, while body functions and structures include all human body parts and their functions. This study focused on sensory profiles and sleep quality of adults with LD. These components appear in the ICF classification in the section of body functions [20].

### Sensory and sleep functions

Sensory modulation describes the complex process of perceiving sensory information and generating responses that are appropriately graded to or are congruent with the situation [21]. The term sensory modulation disorder (SMD) recently received a diagnostic code by the Interdisciplinary Council on Developmental and Learning Disorders [22]. Clinically, persons with SMD can present as hypo-responsive or hyper-responsive, or have labile reactions to sensation [23]. SMD are hypothesized to result from a disrupted nervous system processing of sensory stimuli [24]. The prevalence of sensory processing disorders in children in the general population was recently estimated as 5.3% [25], and significant implications of these disorders have been noted in every area of daily activities, academic function, play and leisure, and in habits and routines as well. [22]. No data regarding the prevalence of sensory disorders among adults could be found for this study. However, it is important to note that difficulties in auditory, visual and phonological processing of stimuli among the population with dyslexia have been widely reported [26], as have difficulties in tactile perception among adults with dyslexia [27].

Poor sleep refers mainly to insomnia which, according to the DSM-IV, is a complaint of difficulty initiating and/or maintaining sleep and/or non-restorative sleep, occurring at least three times per week and lasting over three months, with significant daytime consequences [28]. The prevalence of chronic insomnia in Western countries is estimated as 10% of the adult population and between 19% [29] and 40% experiencing occasional insomnia [30, 31]. Recent evidence has supported the hypothesis that sleep plays a key role in processes of learning and memory [32]. Bruni et al. found disturbances in sleep quality among children with dyslexia compared to normally reading children, and hypothesized a relationship between sleep quality and the severity of reading difficulties [33]. Among adults, low sleep quality was found to be related to daytime difficulties [34], activity impairment, low work productivity [35] and low health-related measures of QoL [36, 37].

The literature reveals relationships between sensory and sleep functions [28]. In his review, Velluti suggested that the central nervous system and sensory input have reciprocal interactions upon which normal sleep/waking cycling and behavior depend [38]. Engel-Yeger and Shochat found that among healthy adults, sleep quality was predicted significantly by hypersensitive patterns in the tactile, visual and auditory modalities [28].

As far as we know, no literature exists concerning sensory disorders and sleep among adults with LD. However, due to the high co-occurrence of LD and ADHD it is important to note that somatosensory processing disorders have been reported among children with ADHD [39, 40, 41], and that sleep disturbances have been widely reported both among children and adults with ADHD [42]. Finally, sleep quality has been found to correlate with sensory modulation patterns among adolescents with ADHD [43].

### Purpose

The aims of this study were: (a) to describe body functions such as sensory modulation and sleep quality of adults with LD, and to compare them with those of a matched control sample, in light of socio-demographic characteristics of adults with LD; (b) to analyse the relationships between these body functions in the entire sample and in each group. The results of the study will serve to demonstrate the complicated health picture of adults with LD and the need to relate to this population by means of a comprehensive health perspective such as the ICF model in order to improve their daily activity, participation and QoL.

It is important to note that unlike most of the existing research, which attempts to specify the type of LD they present, in light of the information previously described about the intricacy of LD, in this study all types of specific LD known in the literature were included and generally related to- as LD.

## Methods and Materials

### Study design and administration

This quasi-experimental case-control design study included a convenience sample of 110 adults from the Southern and Central regions of Israel. Data was collected between March 2011 and August 2012. Each participant was met individually by the researcher in a quiet location and signed a written informed consent. Each participant filled a socio-demographic questionnaire followed by an extensive set of evaluations and questionnaires. Adults with LD could ask for the accommodation of having the questions read aloud, and were offered a free professional advisory hour for their participation.

### Ethics statement

The ethics committee for human subject research in the faculty of social welfare and health sciences at Haifa University approved this study in October 2010 under the name "An alternative model for identification of strengths and weaknesses in occupational performance, participation and quality of life among adults with learning disabilities—Construction of a correlated evaluation set and formation of a theoretical model" (confirmation No. 170/10). An informed consent was signed by each participant and afterwards personal subjects' identifiers were kept separately from the data.

### Study population

The final sample size of 110 participants (55 adults with LD and 55 controls) was determined using the statistical power analysis program described by Faul, Erdfelder, Land, and Buchner [44]. The G*Power3 program calculated a critical t of 1.659 with 108 df and a sample power of 0.831 for 21 central constructs which were examined with an effect size of 0.5 and α error probability of 0.05.

The inclusion criteria were: 20–50 years of age, with Hebrew reading and writing at the level of mother tongue, intact vision and hearing or corrected with an aid, without motor or neurological disabilities, generally healthy with no chronic diseases or significant injuries which may influence daily activities and QoL. Participants of the study group had to present a formal professional evaluation which indicated that they were diagnosed as having a LD. Controls had to answer "no" to both questions: "Have anyone ever told you that you may have a LD?" and "Did you ever think you may have a LD?". Possible confounding variables as gender, age, level of education and socio-economic status were controlled by a procedure of group matching.

Based on the documents presented by the participants, three main types of specific LD were included within the study group (n = 55): dyslexia, dysgraphia and dyscalculia. Descriptive statistics revealed that thirty-three participants (60%) had dyslexia; 31 (56%) had dysgraphia; and 22 participants (40.7%) had dyscalculia. Among the study group, twenty-two participants (40.7%) had only one out of those specific LD, 21 participants (38.9%) had a combination of two specific LD, and 11 participants (20.4%) presented a combination of all these types of specific LD. In addition, difficulties in attention were reported among 42 participants (77.8%) in this group.

### Outcome measures

The following instruments were used to examine socio-demographic characteristics and sensory and sleep functions among adults with LD and their controls:

**Socio-demographic questionnaire**. A 36-question self-report questionnaire which was constructed for this study. Twenty-five questions related to socio-demographic information of the participant. Eleven additional questions related to his past experiences in high-school, and employment and developmental background.

**Sensory Profile—Adolescents/Adult version (AASP)** (Hebrew version) [45]. A 60 item self-report questionnaire aimed to examine sensory modulation and processing of the subject. Using a five-point Likert scale, participants indicate how often they respond to a sensory event in the manner described in each item. For scoring, the 60 items are sorted into four subscales—low registration, sensation seeking, sensory sensitivity and sensation avoiding (based on factor analysis)—reflecting different sensory processing patterns. The questionnaire is standardized, was reported to have good internal consistency psychometric properties [46] and has norms for the general population aged 18–64 [45].

**Mini Sleep Questionnaire (MSQ)** (Hebrew version) [47]. A short 10 item self-report questionnaire. The MSQ is aimed to examine the subject's sleep quality and risk for insomnia. Participants indicated how often they face different sleep difficulties on a seven-point scale. Two scores were calculated: a mean of the 10 items for a final score of sleep quality, and an insomnia score, calculated by summing items 1, 2 and 7. The Hebrew version was reported to have discriminant-validity between adults with and without post-traumatic-stress-disorder [48], and sensitive to change in sleep quality following intervention among women who suffered from headaches [49]. In the current study two participants in the adults with LD group missed items in the MSQ questionnaire, therefore their data is missing for some of the statistical analysis.

### Statistics

Descriptive statistics were calculated for socio-demographic characteristics. Chi-tests, Mann-Whitney tests and Kolmogorov-Smirnov tests were conducted to examine between-groups differences in each of the calculated subscales scores and grades for every instrument. Pearson correlations and regression predictive models were conducted to examine relationships between the body functions.

## Results

### Socio-demographic characteristics

Table 1 demonstrates that subjects of the adults with LD group (n = 55) and controls (n = 55) were matched by gender, average age, level of education, and socio-economic status. Even though, Chi-tests revealed significant differences between the groups in some socio-demographic characteristics, significantly more adults with LD attended more than one high-school, were single and had no children compared to controls.

**Table 1:** Socio-demographic characteristics of the adults with LD group and their matched control group.

Socio-demographic characteristics		LD	Control	χ²	P-value
Gender	Males	34.5%	23.6%	1.587	0.208
	Females	65.5%	76.4%		
Age (years)	X(SD)	29.58(6.4)	31.18(6.4)		0.133^1^
	Range (years)	20–46	23–47		
Level of Education	Elementary	1.8%	0%	2.885	0.577
	High school	47.3%	41.8%		
	Technical	7.3%	3.6%		
	1^st^ Degree	25.5%	27.3%		
	Advanced degrees	18.2%	27.3%		
S-E-S*	1	58.2%	43.6%	2.921	0.232
	2	25.5%	40%		
	3	16.4%	16.4%		
One High School	Yes	74.5%	90.9%	5.153	.021
	No	25.5%	9.1%		
Family Status	Single	63.6%	21.8%	20.403	.001
	Dating	3.6%	10.9%		
	Lives with partner	7.3%	10.9%		
	Married	3.6%	7.3%		
	Married + Children	20.0%	47.3%		
	Divorced	1.8%	1.8%		
Number of Children	No children	78.2%	49.1%	12.157	.016
	One child	5.5%	9.1%		
	Two children	5.5%	21.8%		
	Three or more children	10.9%	20%		

*S-E-S was classified as follows: 1 = low income per person for a single participant or per home for a married participant; 2 = average income; 3 = higher than average income.

^1^ Mann-Whitney test.

### The AASP

The percentage of subjects with different sensory processing levels (lower, similar to most people, or higher) in both groups were compared in each of the subscales of the AASP. Table 2 shows that the Chi-tests revealed significant differences between the groups in all four subscales. In the adults with LD group fewer participants than in the control group demonstrated sensory profiles that are similar to most people in the general population. Compared to controls, significantly more adults with LD had higher levels of low registration, sensory sensitivity and sensory avoiding and significantly more adults with LD had lower levels of sensory seeking.

**Table 2:** Sensory processing levels in each group for the four AASP subscales scores (percentages).

AASP subscales	Lower than most people %		Similar to most people %		Higher than most people %		χ²	P-value
	LD	Control	LD	Control	LD	Control		
Low registration	3.6	12.7	40	80	56.4	7.3	30.940	<.001
Sensory seeking	29.1	9.1	60	81.8	10.9	9.1	7.699	0.021
Sensory sensitivity	1.8	3.6	34.5	81.8	63.6	14.5	27.849	<.001
Sensory avoidance	9.1	10.9	56.4	81.8	34.5	7.3	12.452	0.002

The AASP subscales scores did not meet assumptions for a MANOVA test. Therefore, Kolmogorov-Smirnov tests were used. As shown in Table 3, adults with LD obtained significantly higher scores than controls in three out of the four subscales.

**Table 3:** Means and standard deviations of AASP subscales scores in both groups.

AASP subscales		LD	Control	P-value^1^
Low registration	X(SD)	36.53 (7.79)	29.14 (5.24)	<.001
	Range	19–55	18–45	
Sensory seeking	X(SD)	46.98 (7.33)	48.78 (5.99)	.141
	Range	29–62	28–62	
Sensory sensitivity	X(SD)	43.60 (8.82)	35.98 (5.62)	<.001
	Range	24–59	23–52	
Sensory avoidance	X(SD)	38.21 (8.33)	33.74 (5.72)	.004
	Range	24–57	22–48	

^1^ Kolmogorov-Smirnov test.

### The MSQ

As shown in Table 4, a Kolmogorov-Smirnov test demonstrated significant differences between the groups for both the final score of sleep quality and for the MSQ insomnia score. Adults with LD had significantly lower sleep quality and more tendencies for insomnia than controls.

**Table 4:** Means and standard deviations of MSQ scores in both groups.

MSQ scores*	LD	Control	p-value^1^
Sleep quality X(SD)	3.10 (0.77)	2.38 (0.55)	<.001
Insomnia X(SD)	10.40 (3.27)	8.14 (2.51)	.019

*Higher grades indicate lower sleep quality/more tendencies for insomnia.

^1^ Kolmogorov-Smirnov test.

### Correlations between AASP subscale scores and sleep quality

Sensory sensitivity correlated significantly with sensory avoiding in both groups. In the control group this was a low correlation (r = 0.39, p = .003), and in the adults with LD group this was a medium-high correlation (r = 0.70, p < .001). In addition, in the control group the MSQ final score significantly correlated only with sensory sensitivity (r = 0.30, p = .027), while in the adults with LD group the MSQ final score was significantly correlated with sensory sensitivity (r = 0.32, p = .023) and with low registration (r = 0.45, p = .002).

### Linear regression analysis

The results presented in Tables 5 and 6 indicate that the group (N = 108) accounted for 22.7% of the variance of sleep quality as reflected in the MSQ final score (F [1,106] = 31.192, β = 0.477, p < .001). Two regression models were calculated to predict sleep quality among the entire sample: (a) The subscale score of sensory sensitivity added 7.5% of prediction (F [2,105] = 22.768, β = 0.317, p < .001) (Table 5) meaning that sensory sensitivity accounted for 7.5% of the variance in sleep quality above group membership (adults with LD versus controls); (b) The subscale score of low registration added 10.2% of prediction (F [2,105] = 25.807, β = 0.364, p < .001) (Table 6) meaning that low registration accounted for 10.2% of the variance in sleep quality above group membership. Additional regression analysis was calculated for each group separately due to the differences which were found between the groups in the correlations between the body functions. In the control group (n = 55), the sensory sensitivity subscale score accounted for 8.8% of the variance in sleep quality (F [1,53] = 5.141, β = 0.297, p = 0.027). However, in the adults with LD group (n = 53) the low registration subscale score accounted for 19.9% of the variance in sleep quality (F [1,51] = 12.633, β = 0.446, p < .001).

**Table 5:** Sleep quality prediction by group membership and sensory sensitivity subscale score.

	Model 1			Model 2		
Variable	B	SE B	β	B	SE B	β
Group	.722	.129	.477	.481	.143	.318
Sensory sensitivity				.309	.092	.317
R² change	.227			.075		
F change in R	31.192*			22.768*		

* p <. 001

**Table 6:** Sleep quality prediction by group membership and low registration subscale score.

	Model 1			Model 2		
Variable	B	SE B	β	B	SE B	β
Group	.722	.129	.477	.459	.138	.303
Low registration				.368	.092	.364
R² change	.227			.102		
F change in R	31.192*			25.807*		

* p < .001

## Discussion

The current study was aimed at describing body functions such as sensory modulation and sleep quality of adults with LD and comparing them with a matched control sample with respect to the socio-demographic characteristics of adults with LD. This study also aimed to analyse relationships between these body functions in the entire sample and in each group.

Some interesting results were noticeable from the socio-demographic features of adults with LD. A significantly higher percentage of adults with LD attended more than one high-school, were single and had no children compared to their matched controls. These findings may expose limitations in the activity and participation in other life areas among adults with LD in addition to their academic difficulties which are well recognized and discussed in the literature.

The sensory profiles of adults with LD were significantly different from those of controls. While the controls demonstrated sensory profiles similar to the general norms [45], this was not the picture among adults with LD. In the adults with LD group fewer participants than in the control group demonstrated sensory profiles similar to those of most people in the general population: significantly more adults with LD had higher levels of low registration, sensory sensitivity and sensory avoiding and lower levels of sensory seeking. These results indicate that *the sensory functions of adults with LD differ* from those of controls.

According to Dunn's model of sensory processing, which constitutes the conceptual framework of the AASP questionnaire, low registration refers to high thresholds with passive responding strategies, sensory sensitivity refers to low thresholds with passive responding strategies, sensory seeking refers to high thresholds with active responding strategies, and sensory avoiding refers to low thresholds with active responding strategies. The daily meaning is that persons with low registration do not notice sensory events in daily life that are readily noticed by most people, those with sensory sensitivity notice more sensory stimuli than most people and therefore are easily distracted, sensory seekers actively look for sensory input and sensory avoiders actively find ways to limit sensory input throughout the day. For example, they stay away from distracting settings, create rituals for daily routines and become unhappy when these rituals are disrupted [50].

Sensory disorders co-occurred amongst adults with LD significantly more than among controls. These results are found to be in line with reports in the literature regarding difficulties among the population with dyslexia in specific sensory functions as auditory, visual and phonological processing [26] as well as in tactile processing [27] and, more specifically, in the processing of rapid stimulus sequences [51]. Further investigation is required on the relationships between specific sensory patterns (i.e., low registration, sensory sensitivity, sensory seeking, sensory avoiding), specific LD (i.e. dyslexia, dyscalculia, dysgraphia) and specific academic skills (i.e. listening, thinking, speaking, writing, spelling, mathematical calculating). In addition, deficits in sensory functions have been reported to have ramifications in daily activity and participation among children in general [22] and, more specifically, in the participation of children with ADHD in leisure activities [52]. Therefore, in accordance with the ICF model, it is recommended to further investigate the characteristics of daily activity and participation of adults with LD in various life areas related to their sensory profile.

Further to different sensory features adults with LD also demonstrated lower sleep quality than controls, and more tendencies towards insomnia. These results indicate that *the sleep quality of adults with LD also differs* from those of controls. Previous literature indicated that among healthy adults poor sleep quality was associated with diminished attention and executive functions [53] and that sleep deprivation was associated with declined performance in various cognitive tasks [54]. The high co-occurrence of sleep problems among adults with LD as found in this study requires further studies concerning the relationships between sleep and academic functioning among the population with LD. In addition, sleep disorders have been reported as associated with depression and anxiety disorders [55], activity impairment, low work productivity [35] and low health-related QoL [36]. Since the results of the current study exhibited that adults with LD constitute a risk group for these consequences, further studies on such ramifications on the lives of adults with LD are required.

Different correlation levels were found between the AASP subscale scores in each of the groups. In both groups, sensory sensitivity correlated significantly with sensory avoiding. However, in the adults with LD group these relationships were stronger. As mentioned above, sensory avoiders actively find ways to limit sensory input throughout the day. Furthermore sensory avoiding has been associated with fear, negative affect and neuroticism [50]. In concurrence with the ICF concepts, more studies are required on whether adults with LD avoid activities and participation in various life areas. Whether different sensory profiles as measured in the current study may explain depression and anxiety disorders which were reported in high percentages among the population with LD should also be examined.

Differences were found between the groups in the correlations between their AASP subscale scores and their sleep quality. While sleep quality for controls was correlated with sensory sensitivity only, sleep quality for adults with LD was correlated with both sensory sensitivity and low registration. The low registration score was a better predictor of sleep quality than the sensory sensitivity score for the entire sample. Using a separate predicting model for each group, it was found that the sensory sensitivity in the control group was, indeed, the significant predictor of sleep quality as previously reported among healthy adults [28]. However, for adults with LD the low registration subscale score accounted for 19.9% of the variance in sleep quality, and was a more significant predictor of sleep quality than sensory sensitivity. It is assumed that in this study, low registration was a better predictor of sleep quality for the entire sample as well, due to the high representation of adults with LD (50%). These results indicate that *adults with LD are a unique population with diverse body functions and unique relationships between their body functions*.

Gijsbers Van Wijk and Kolk suggested that both too many and too few external cues can lead to the need to invest increased attention to internal cues and, therefore, to higher rates of fatigue [56]. However a previous study revealed that low registration did not differentiate between good and poor sleepers among healthy adults [28]. Therefore, it is suggested that *low registration may be a discriminant sensory characteristic of adults with LD*. Further studies are required to examine this possibility. However, if such possibility is proved in the future, due to the paucity of literature on sensory and sleep functions among adults generally, and among adults with LD specifically, two questions remain open. One is whether low registration of sensory stimuli among adults with LD may have some relationships with their specific academic deficits due to not noticing sensory stimuli which are noticeable to other people. Further studies on the unique relationship of the low registration sensory pattern with other health characteristics and body functions of adults with LD are required in order to gain further understanding of such a possible mechanism. The second question is whether the low sensory registration among adults with LD influences their sleep quality which, in turn, may cause secondary problems in academic achievement as well as depression, anxiety and other difficulties in daily activity and participation, and low QoL as described in existing literature among people with low sleep quality. Further investigation in this direction is also recommended.

### Clinical and research implications

The results emphasize the complexity of the health condition of adults with LD and indicate that there is a need to relate to this population both in clinical settings and in research field through a comprehensive health perspective such as the ICF model. Such a perspective may contribute to further understand the unique health characteristics and daily needs of this population.

The results demonstrate that sensory and sleep body functions of adults with LD need to be considered in clinical settings. Therefore, additional research on these functions and the relationship between them should be performed. Research on other body functions of this population is also recommended due to the findings in this study which indicate that this population has its own unique characteristics.

Furthermore, according to the ICF it is suggested that the relationships between different sensory processing patterns and specific LD be examined in the future. The possible implications of the unique sensory and sleep characteristics on adults with LD activity and participation in various life areas and their health-related QoL should be examined as well.

### Strengths and limitations

The strengths of this study are the quasi-experimental case-control design with a matched control group and the use of acceptable measures which have been used widely in other studies for the evaluation of sensory and sleep functions. The sample power in this study is also acceptable and significant. All findings in the study were coherent and matched previous literature.

The limitations of this study focus mainly on the heterogeneity of the sample. Participants demonstrated professional observations of all types of specific LD (i.e.: dyslexia, dysgraphia, dyscalculia), and some of them had reported additional difficulties in attention. This was required in order to represent the complicated health picture of adults with LD. However it may be reflected in the high variability within this group. Also, the results of this study were obtained through self-reports of the subjects on their daily behaviors and therefore should be further examined in the future with additional measures.

## Conclusions

The ICF model may serve as a suitable framework for the research of adults with LD and may encourage the study of the unique health characteristics and body functions of this population as well as their daily activity and participation in various life areas.

In this study, which was conducted according to ICF concepts, it was revealed that adults with LD are a unique population with diverse sensory and sleep characteristics and different relationships between these body functions than those of the controls with no LD. Adults with LD demonstrated significantly more sensory difficulties and lower sleep quality than controls. Therefore, the sensory patterns and sleep quality of adults with LD need to be considered in clinical settings and in future research, as well as their implications on the daily activity and participation and QoL of adults with LD.

Gaining further understanding of the unique characteristics of adults with LD may improve evaluation and intervention processes among this population and, consequently, contribute to their daily functioning and QoL.
